# Method of Quantifying Size of Retinal Hemorrhages in Eyes with Branch Retinal Vein Occlusion Using 14-Square Grid: Interrater and Intrarater Reliability

**DOI:** 10.1155/2016/1960190

**Published:** 2016-10-27

**Authors:** Yuko Takashima, Masahiko Sugimoto, Kumiko Kato, Maki Kozawa, Kengo Ikesugi, Hisashi Matsubara, Mineo Kondo

**Affiliations:** Department of Ophthalmology, Mie University Graduate School of Medicine, Tsu, Japan

## Abstract

*Purpose*. To describe a method of quantifying the size of the retinal hemorrhages in branch retinal vein occlusion (BRVO) and to determine the interrater and intrarater reliabilities of these measurements.* Methods*. Thirty-five fundus photographs from 35 consecutive eyes with BRVO were studied. The fundus images were analyzed with Power-Point® software, and a grid of 14 squares was laid over the fundus image. Raters were asked to judge the percentage of each of the 14 squares that was covered by the hemorrhages, and the average of the 14 squares was taken to be the relative size of the retinal hemorrhage.* Results*. Interrater reliability between three raters was higher when a grid with 14 squares was used (intraclass correlation coefficient (ICC), 0.96) than that when a box with no grid was used (ICC, 0.78). Intrarater reliability, which was calculated by the retinal hemorrhage area measured on two different days, was also higher (ICC, 0.97) than that with no grid (ICC, 0.86). Interrater reliability for five fundus pictures with poor image quality was also good when a grid with 14 squares was used (ICC, 0.88).* Conclusions*. Although our method is subjective, excellent interrater and intrarater reliabilities indicate that this method can be adapted for clinical use.

## 1. Introduction

Retinal vein occlusion (RVO) is the second most common type of retinal vascular disorder after diabetic retinopathy [1–3]. RVO can be classified into three types according to where the occlusion is located: central retinal vein occlusion (CRVO), branch retinal vein occlusion (BRVO), and hemiretinal vein occlusion (HRVO). Among these, BRVO has the highest incidence with a yearly rate of approximately 1 in 150 000 in the USA [1].

RVOs are usually associated with massive hemorrhage in the sector of the retina drained by the occluded vein. The images obtained by optical coherence tomography (OCT) have shown that the retinal hemorrhages due to a BRVO were located in the intraretinal space or in the subretinal space near the fovea [4–7]. Although it is not completely known whether such intra- and subretinal hemorrhages are toxic to the retinal neurons, an earlier absorption of the hemorrhage would be better because experimental and clinical studies have shown that subretinal hemorrhages can cause damage to the photoreceptors due to the toxic effects of iron and fibrin [8–10]. Recent results suggested that the antivascular endothelial growth factor (anti-VEGF) therapy promoted the regression of retinal hemorrhages in the eyes with a BRVO presumably by the suppression of leakage of fluids from the retinal vessels [11].

Several methods have been proposed to detect and quantify the size of the retinal hemorrhages for different types of retinal diseases [12–17]. In these methods, automated image analysis algorithms of digital color fundus photograph were used. However, these automated programs are not commonly available. In addition, several issues remain including the differentiation of the hemorrhages from retinal vessels and the difficulty in analyzing low-quality fundus images due to cloudy media.

Thus, the purpose of this study was to determine whether a new, simple method can be used to quantify the size of retinal hemorrhages in eyes with BRVO. To accomplish this, we used a grid composed of 14 squares that was laid over the fundus photographs, and three raters estimated the percentage of each square that was occupied by the hemorrhage. We shall show that our method is relatively simple and versatile, and the interrater and intrarater reliabilities were very high.

## 2. Methods

### 2.1. Subjects and Fundus Photographs

This was a cross-sectional, retrospective observational study. Fundus photographs of 35 eyes of 35 consecutive patients with a BRVO who were examined in the Mie University Hospital from April of 2014 to April of 2015 were studied. The color fundus photographs were recorded with a digital fundus camera (TRC-50Dx, Topcon, Japan) with a visual angle of 60 degrees (diameter). Digital images of the macular area with the fovea located at the center were used for the analyses. The fundus images were extracted as JPG files consisting of 3216 × 2136 pixels.

The procedures used conformed to the tenets of the World Medical Association's Declaration of Helsinki. The Mie University Hospital (Tsu, Japan) Institutional Ethics Review Board approved this retrospective observational study using the patients' medical records (#2383). A written informed consent was not given by subjects for their fundus photographs to be used retrospectively in this study, but patient information has been anonymized and deidentified prior to the analyses.

### 2.2. Method of Determining Size of Retinal Hemorrhage in Fundus Photograph

**Figure 1:**
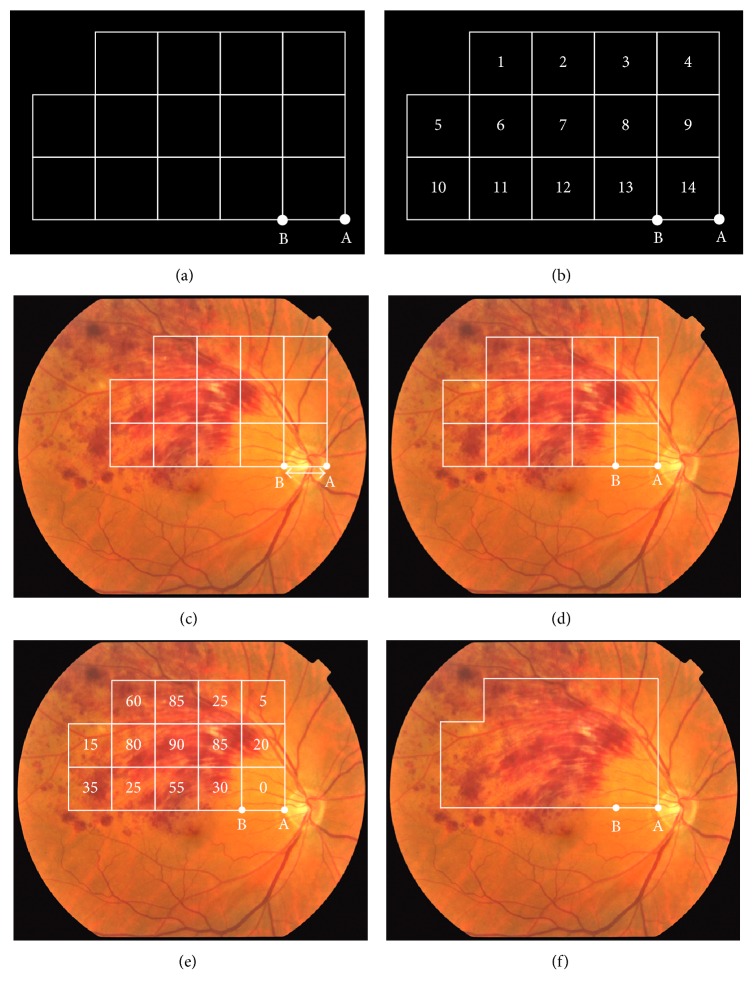
Method of determining the area occupied by a retinal hemorrhage in a fundus photograph using a grid with 14 squares. First, a grid with 14 squares (a) is created by the PowerPoint (PPT) software (a). Each square is numbered starting at the top left (b). Next, a fundus photograph of BRVO was downloaded into a PPT file, and the grid with 14 squares was laid over the fundus photograph. The grid was enlarged or reduced so that one side of a square grid (the length of the line BA) would match the horizontal diameter of the optic disc (c). Then, the point A on the grid was placed at the temporal edge of the optic disc (d). Then, retina specialists (raters) visually estimated what percent of the area of each of the 14 squares was occupied by the retinal hemorrhage. This relative size ranged from 0 to 100% in increments of 5% (e). The total was averaged to yield the size of the hemorrhage as a percent of the entire grid. To determine the effectiveness of using the grid, an empty box of the same size as the grid was created (f), and this box was laid over a fundus photograph in the same way.

To determine the size of the retinal hemorrhages in a fundus photograph, a grid with 14 squares (Figure 1(a)) was created with the PowerPoint (PPT) software. The squares were grouped so that the grid could be easily laid over a fundus photograph. The grid was laid over a fundus photograph with Point A coincident with the temporal edge of the optic disc. Each grid square was numbered starting at the top left (Figure 1(b)).

Next, a fundus photograph from a patient was downloaded to the PPT program on a PC, and the fundus photograph was enlarged to fill the entire monitor screen. Some photographs may be dark overall or of poor contrast depending on the conditions when the fundus photograph was taken. In such cases, the optimal brightness and contrast were chosen so that the retinal hemorrhage was more readily evident in the PPT image. Six adjustable levels of brightness and contrast are present in the PPT program in its default settings, and these levels were used.

Next, the grid with 14 squares was laid over the fundus photograph, and its size of the sides was adjusted so that it equaled the horizontal diameter of the optic disc, line A to B in Figure 1(c). Then, point A on the grid was moved to temporal edge of the optic disc (Figure 1(d)).

Next, three retina specialists determined what percent of the area of each of the 14 squares was occupied by the retinal hemorrhage. The range of the relative sizes was 0 to 100% in 5% increments. If hemorrhage was not evident in a square, the square was rated as 0%, and if a hemorrhage occupied about one-quarter of the area of a square, a rating of 25% was given. If a hemorrhage occupied almost all of the area of a square, a rating of 100% was given. The ratings did not assess the depth of the hemorrhage as displayed by the color and only how much of the square the hemorrhage occupied was determined (Figure 1(e)).

After all of the squares had been graded, the percentage of each square that was occupied by the hemorrhage was totaled for all 14 squares. This total was averaged to yield the area of the hemorrhage as a percentage of the entire grid.

To determine the effectiveness of using the grids for the visual assessment, a box of the same size but without the grid was created (Figure 1(f)). This box was laid over the same area of the fundus photograph, and the same raters were asked to estimate the percentage of the entire box that was occupied by the hemorrhages. This value similarly ranged from 0 to 100% in increments of 5%.

### 2.3. Interrater Reliability

To examine the degree of reliability, three retina specialists estimated the size of the retinal hemorrhages occupying each square in the fundus photographs. The raters used either the grid (Figure 1(d)) or the box with no grids (Figure 1(f)). Fundus photographs of 30 BRVO eyes whose image quality was good were used. To compare the interrater reliability of the two methods of measuring the size of the retinal hemorrhage area, the intraclass correlation coefficient (ICC) was calculated for the three raters. The ICC was calculated when the grid with 14 squares and when the box with no grid were used. The two values were compared. The ICC was calculated with the SPPS Statistics Version 23.

### 2.4. Intrarater Reliability

To examine the degree of intrarater in determining the size of the retinal hemorrhages, the same three raters measured the size of the retinal hemorrhages on the same 30 fundus photographs one month after their initial measurements. The ICC was calculated when raters used the grid with 14 squares and when they used the box with no grid, and the two values were compared.

### 2.5. Analyses of Size of Retinal Hemorrhages in Poor Quality Fundus Photographs

To determine whether the size of retinal hemorrhages could be quantitatively determined when the fundus photographs were of poor image quality. Accordingly, five poorer quality fundus photographs were selected from the 35 BRVO fundus photographs. The poor quality was mainly due to poor mydriasis or cataract. The grid with 14 squares was used to measure the size of the hemorrhages. Measurements were done by the same three raters, and the ICC was calculated to determine the interrater reliability for the three raters.

### 2.6. Quantitative Determination of Changes in Size of Hemorrhages with Time

Fundus photographs of one eye with BRVO (#21) were used to determine the changes in the size of the retinal hemorrhages with increasing time. The photographs at the initial visit and at 2, 4, 6, 8, and 10 months later were used. The three raters used the grid with 14 squares to measure the relative size of the retinal hemorrhages. The changes in the size of the hemorrhages (%) were plotted against time.

## 3. Results

### 3.1. Clinical Characteristics of BRVO Patients

The age of 35 consecutive patients with a BRVO (18 men and 17 women) ranged from 38 to 84 years. The decimal best-corrected visual acuity (BCVA) ranged from 0.1 to 1.0 with a median of 0.4. The average interval between the onset of symptoms and the taking of the photographs was 6.7 weeks with a range of 1 to 52 weeks.

### 3.2. Interrater Reliability

**Figure 2:**
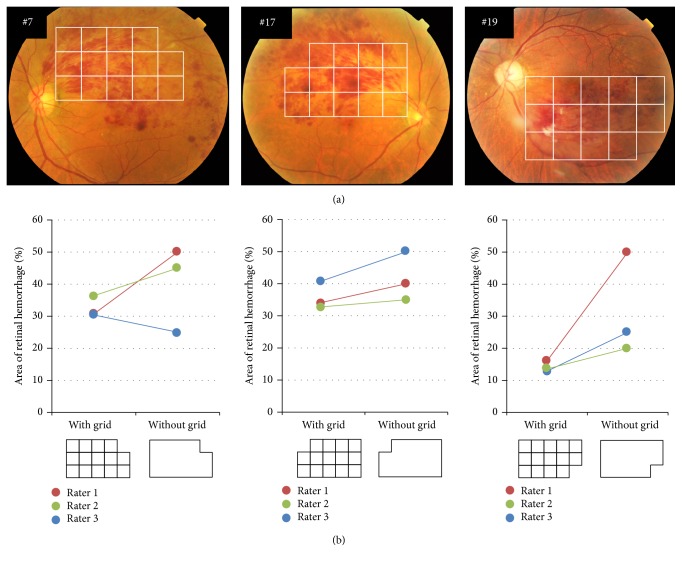
Three representative fundus photographs of eyes with a BRVO, and the results of the retinal hemorrhage sizes measured by using the grid with 14 squares and an empty box with no grid. (a) shows three fundus photographs of eyes #7, #17, and #19 that are overlaid with the grid to measure the size of the retinal hemorrhages. (b) is a graph plotting the retinal hemorrhage size measured with the 14-square grid and with the empty box made by the three raters. Note that there is less variation between three raters when they used a grid with 14 squares than when they used the empty box.

The interrater reliabilities for the three raters for three typical BRVO eyes (#7, #17, and #19) are shown in Figure 2.  Figure 2(a) shows the fundus photographs overlain with the grid that was used to estimate the size of the retinal hemorrhage, while Figure 2(b) shows a graph of the size of the hemorrhage estimated by the three raters. The size of the hemorrhages when the grid was used and when the box with no grid was used is shown. There was a higher reliability when the grid was used than when the empty box was used.

The area of the hemorrhage was measured in all 30 eyes with BRVO by the three raters, and the measurements obtained using both methods were statistically analyzed (Table 1). The ICC was 0.96 when the grid was used and 0.78 when the empty box was used. Thus, the interrater reliability was higher when the grid with 14 squares was used.

### 3.3. Intrarater Reliability

The intrarater reliability when the area of hemorrhage was measured by the grid with 14 squares was used was 0.98, 0.98, and 0.95 for the three raters (Table 2). When the empty box with no grid was used, the ICC for each of the three raters was 0.85, 0.84, and 0.89. These findings indicate that the use of the grid resulted in a higher intrarater reliability.

### 3.4. Size of Hemorrhages in Poor Quality Fundus Photographs

**Figure 3:**
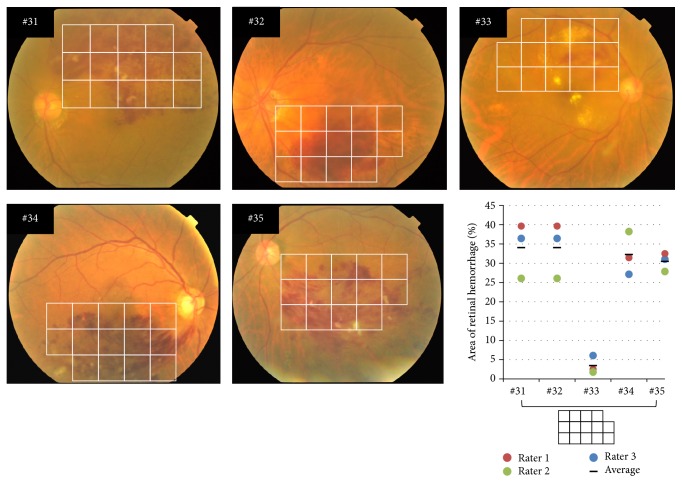
Five fundus photographs with poor quality, and the results of retinal hemorrhage sizes measured by using grid with 14 squares. The grid with 14 squares was laid over five fundus photographs of poor quality (#31–#35). The most right panel at the bottom is a graph plotting the retinal hemorrhage size measured using the grid with 14 squares by the three raters and averaged values.

Quantitative determinations of the size of the hemorrhages in five poor quality fundus photographs are shown in Figure 3. The five fundus photographs were overlain with a grid to measure the area of the retinal hemorrhage (#31–#35), and a graph of the area of retinal hemorrhage measured by three raters (right column of lower panel) is shown. The ICC for three raters for these five photographs was 0.89. This value was lower than that when fundus photographs with higher quality were used at 0.96 (Table 1). However, these findings demonstrated that highly reliable estimates could still be made even with poor quality fundus photographs.

### 3.5. Assessment of Changes in Size of Hemorrhages in Fundus Photographs over Time

**Figure 4:**
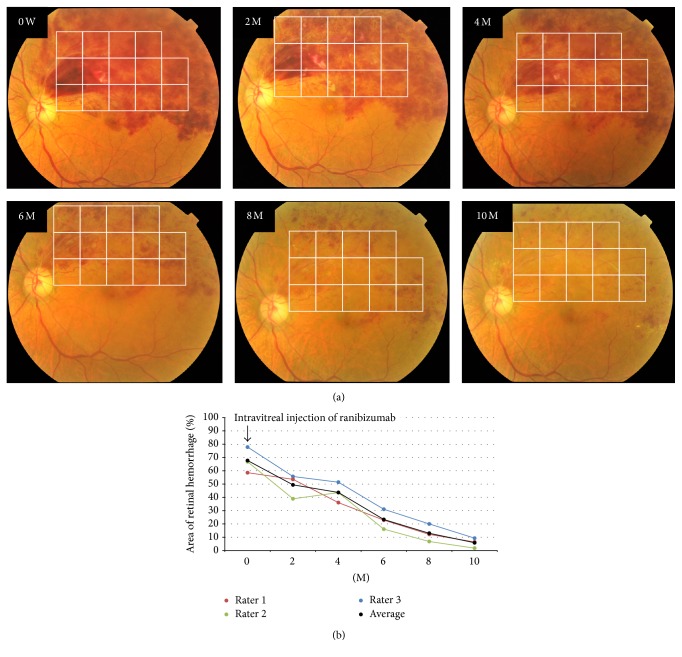
Changes in the retinal hemorrhage area over the time in an eye with BRVO. The retinal hemorrhage area was measured by the three raters using a grid with 14 squares at the initial visit (0) and at 2, 4, 6, 8, and 10 months after the initial visit. Intravitreal ranibizumab was injected at the initial visit. Fundus photographs at each time point are shown in (a), and values of retinal hemorrhage size measured by each three raters and averaged values are plotted in (b).

The changes in the size of the hemorrhages from the initial visit to ten months later in a typical patient with BRVO are shown in Figure 4(a). The sizes of the hemorrhage area using the grid with 14 squares by the three raters (red, green, and blue) and averaged values (black) are plotted in Figure 4(b). A single Intravitreal injection of ranibizumab was performed at the initial visit to treat the macular edema (arrow), and macular edema gradually improved without any recurrence. The mean area of the retinal hemorrhage was 67.7% at the initial visit, which decreased gradually to 5.8% at 10 months after the initial visit.

## 4. Discussion

The results showed that the size of the retinal hemorrhages measured by three raters had a high interrater reliability with an ICC of 0.96 which was higher than that when a box with no grid was used with an ICC of 0.78 (Table 1). In addition, the mean intrarater reliability was significantly higher when the grid was used at an ICC of 0.97 than when the box with no grid was used at an ICC of 0.86 (Table 1). These results indicate that this method is reliable and should be able to be adapted for clinical use. For example, it can be used to follow the regression of the hemorrhage after anti-VEGF therapy [11].

There are several advantages in our method of determining the size of the retinal hemorrhages in the fundus photographs of eyes with BRVO. The first advantage is that this method is simple and highly flexible. If a PC with a software such as PPT is used, the size of hemorrhages can be measured by anyone. The size can be measured in photographs of any resolution and any format. In addition, the size of the hemorrhages can be measured even in a printed fundus photograph by scanning the photograph into a PC.

The second advantage is that the size of the retinal hemorrhages can be measured even if different fundus cameras are used to photograph the same region. The current method decides the size of the region to be measured based on the horizontal diameter of the optic disc. If a fundus photograph is taken from the same patient, the same region can be measured even if different fundus cameras were used. If, for example, a facility changes its fundus camera during the follow-up examinations, the same region can still be accurately measured. In addition, if the region recorded in a fundus photograph is slightly shifted due to the changes in the fixation, the current method allows the movement of the grid to the region where the initial measurements were made.

The third advantage of the current method is that the area of a hemorrhage can be measured even if the quality of the fundus photograph is relatively poor. Fundus photographs from elderly patients can often be of poorer quality due to poor mydriasis or a cataract. However, the current method is subjective, so the area of the hemorrhage can be determined even if image quality is poor. When the current method was used to determine the area of the hemorrhage in poor quality fundus photographs of five eyes, the ICC for three raters was still high at 0.89 (Figure 3).

Conversely, the current method has several disadvantages. The first disadvantage is that the size of the hemorrhages is estimated subjectively, so it is influenced by the experience of the rater. To overcome this drawback, using the mean value of retinal hemorrhage determined by several raters could improve the accuracy.

The second disadvantage is that only a limited region can be measured. The current method of using a grid with 14 squares can measure the area of most of the posterior pole, which is where retinal hemorrhage commonly occurs in eyes with BRVO. However, the area of hemorrhage in the region outside the grid box cannot be determined. Although the surrounding retina could be covered by increasing the number of squares, some of the squares may extend beyond the fundus photographs. This is why the area measured was designed as shown in Figure 1(d).

The third disadvantage is that the length of the side of a square matched the horizontal diameter of the optic disc. Thus, the region being measured depended largely on the size of the optic disc. The horizontal diameter of the optic disc was used so that the same site could be measured in a patient regardless of the view angle of the camera when the photograph was taken. However, this restricts the region that can be measured as when a patient with a small optic disc is examined. Other methods of deciding the region to measure besides the size of the optic disc should probably be considered.

In conclusion, we have presented a new method of subjectively determining the size of the retinal hemorrhages in eyes with BRVO. Dividing the fundus into a grid with 14 squares allowed reliable estimates of the sizes of the hemorrhagic areas. The results indicated that this method has a high level of interrater and intrarater reliability. This method can be used to follow the changes in the sizes of the retinal hemorrhages over time (Figure 4), and it could also be used to determine whether the therapy prescribed was effective in reducing the size of the hemorrhagic areas [11].

## Figures and Tables

**Table 1:** Fundamental statistics and interrater reliability for the areas of retinal hemorrhage measured by using a grid with 14 squares and empty box.

	Sample size	Grid with 14 squares			Empty box		
		Avg.	SD	SE	Avg.	SD	SE
Rater 1	30	31.27	20.27	3.70	43.50	22.48	4.10
Rater 2	30	29.10	18.66	3.40	43.33	20.69	3.78
Rater 3	30	28.50	18.27	3.33	38.83	18.23	3.33
Sum	90	29.63	18.91	1.99	41.89	20.42	2.15
ICC		0.96			0.78		

Avg., average; SD, standard deviation; SE, standard error; ICC, intraclass correlation coefficient.

**Table 2:** Intrarater reliability for the area of retinal hemorrhage measured by using a grid with 14 squares and empty box.

	Grid with 14 squares	Empty box
ICC of Rater 1	0.98	0.85
ICC of Rater 2	0.98	0.84
ICC of Rater 3	0.95	0.89

Avg.	0.97	0.86

Avg., average; ICC, intraclass correlation coefficient.
